# Association between a novel four-location outer retinal morphology score and visual outcomes in full-thickness macular hole

**DOI:** 10.1186/s40942-026-00912-4

**Published:** 2026-07-31

**Authors:** Mitsuru Otsubo, Tomoko Ro-Mase, Shun Konno, Ami Konno, Harumasa Yokota, Taiji Nagaoka

**Affiliations:** Department of Ophthalmology, Asahikawa Medical University, 2-1-1-1 Midorigaoka Higashi, Asahikawa, Hokkaido 078-8510 Japan

**Keywords:** Macular hole, Full-thickness macular hole, Optical coherence tomography, Outer retina, Outer retinal morphology, Visual prognosis, Vitrectomy

## Abstract

**Purpose:**

To evaluate the association between a novel four-location outer retinal score (OR score) and postoperative best-corrected visual acuity (BCVA) in eyes with full-thickness macular hole.

**Methods:**

This retrospective study included eyes undergoing vitrectomy for full-thickness macular hole. Preoperative optical coherence tomography (OCT) was used to assess outer retinal morphology at the macular hole edge in four lacations (temporal, nasal, superior, and inferior) using horizontal and vertical scans. Each location was graded, and the sum was defined as the total OR score. Associations with postoperative BCVA at 3 months were evaluated using Spearman’s correlation and multivariable linear regression.

**Results:**

A total of 48 eyes from 48 patients were included in this study. Four eyes in which primary macular hole closure was not achieved after the initial surgery were excluded from the final analysis of postoperative BCVA at 3 months. The OR score was significantly correlated with postoperative BCVA (ρ = 0.458, *p* = 0.0018). However, after adjustment for minimum hole diameter, it was not an independent predictor. The OR score was significantly correlated with minimum hole diameter (ρ = 0.522, *p* < 0.001). Neither the Gass classification nor single cross-sectional OCT classification showed significant associations with postoperative BCVA.

**Conclusions:**

The four-location OR score was associated with postoperative BCVA and may reflect the severity of outer retinal damage. Its lack of independent predictive value suggests that it captures morphological severity overlapping with minimum hole diameter.

**Supplementary Information:**

The online version contains supplementary material available at https://doi.org/10.1186/s40942-026-00912-4.

## Introduction

Macular hole is a vision-threatening condition caused by vitreofoveal traction, leading to significant visual impairment, and vitrectomy is an effective treatment for achieving anatomical closure [1–3]. With the introduction of the inverted internal limiting membrane (ILM) flap technique, high closure rates have been reported even in large macular holes [4–7].

Traditionally, the Gass classification has been used to categorize macular holes. Although this classification, based on slit-lamp biomicroscopy, has contributed to understanding disease progression, it has limitations in terms of objectivity and quantitative assessment. With advances in optical coherence tomography (OCT), detailed evaluation of retinal microstructure has become possible, enabling more objective and quantitative assessment of macular hole morphology [3, 8].

Various OCT-based prognostic factors have been reported. In particular, the minimum hole diameter is considered a key parameter strongly associated with postoperative visual acuity [9]. In addition, preoperative disruption of the external limiting membrane (ELM) and the ellipsoid zone (EZ) has been shown to affect postoperative retinal function, and the postoperative restoration of these structures is also associated with visual outcomes [10–14].

Recently, Pecaku et al. proposed a novel classification based on outer retinal morphology at the macular hole edge using spectral-domain OCT, adapted from outer retinal classifications originally developed for rhegmatogenous retinal detachment, and reported its association with postoperative visual acuity [15–17]. However, this classification is based on a single cross-sectional image and may not fully capture localized structural variations around the macular hole. Recent studies have suggested that macular hole formation may begin with an inner foveal tear, often originating from the temporal side, indicating that the pathological process is not uniform across the entire circumference of the hole [18]. Furthermore, macular hole diameter has been reported to vary depending on the direction of the OCT scan [19]. In addition, in other macular disorders such as lamellar macular hole and macular pseudohole, the detection of morphological features has been shown to depend on the OCT scan pattern [20]. These findings suggest that evaluation using multiple OCT scan orientations may provide a more accurate understanding of macular hole morphology. Because the outer retina remaining at the macular hole edge is likely to reflect structural damage, the severity of outer retinal disruption may vary among different locations around the macular hole. Therefore, evaluation based on a single horizontal scan may not adequately represent the overall severity of outer retinal disruption. Although assessment using multiple B-scans or volumetric OCT could provide more comprehensive information, such approaches are less practical in routine clinical practice. We therefore evaluated the four macular hole edge locations using the conventional horizontal scan together with the orthogonal vertical scan.

In this study, we applied the classification proposed by Pecaku et al. and evaluated outer retinal findings at the macular hole edge in four locations (temporal, nasal, superior, and inferior) using both horizontal and vertical OCT scans. Each region was scored, and the sum was defined as the total outer retinal (OR) score. The purpose of this study was to investigate the association between this novel score and postoperative best-corrected visual acuity (BCVA) and to clarify its clinical significance.

## Methods

### Study design and participants

This retrospective study included patients with idiopathic full-thickness macular hole of Gass stage 2 or higher who underwent vitrectomy at Asahikawa Medical University Hospital between September 2023 and December 2025. The study was approved by the Institutional Review Board of Asahikawa Medical University (approval number: 24072) and adhered to the tenets of the Declaration of Helsinki.

Eyes with uveitis, glaucoma affecting central vision, age-related macular degeneration, proliferative diabetic retinopathy, or diabetic maculopathy were excluded. Highly myopic eyes with macular chorioretinal atrophy or posterior staphyloma were also excluded.

### OCT evaluation and outer retinal classification

Preoperative OCT images were obtained using spectral-domain OCT (SPECTRALIS OCT; Heidelberg Engineering, Heidelberg, Germany). Horizontal and vertical scans were used to evaluate the macular hole edges at four locations: temporal, nasal, superior, and inferior. The proposed OR score was derived from these four evaluated locations obtained from two OCT scan orientations (horizontal and vertical).

Outer retinal findings were classified into four stages (A–D) according to the classification proposed by Pecaku et al. Stage A was defined as preservation of the ELM, EZ, and interdigitation zone (IZ). Stage B was characterized by a hyperreflective homogeneous thickened band representing the photoreceptor inner and outer segments. Stage C showed patchy loss of photoreceptors with a moth-eaten appearance of the outer retina. Stage D indicated severe or complete loss of photoreceptor inner and outer segments, leaving a bare ELM [17].

Two independent graders (M.O. and T. R.-M.) evaluated the OCT images. Interobserver agreement for outer retinal classification was assessed using the weighted Cohen’s kappa coefficient. In cases of disagreement, a consensus was reached through discussion.

Each stage (A–D) was converted to a numerical score (1–4), and the sum of the four locations was defined as the total OR score.

### Surgical procedures

All surgeries were performed using the Constellation Vision System (Alcon, Fort Worth, TX, USA) with either a 25-gauge or 27-gauge system by experienced vitreoretinal surgeons (M.O., S.K., A.K., H.Y., and T.N.).

In cases with coexisting cataract, combined phacoemulsification and intraocular lens implantation was performed. After core vitrectomy, the ILM was stained using 0.025% brilliant blue G. ILM peeling or the inverted ILM flap technique was performed at the surgeon’s discretion. Fluid–air exchange was followed by injection of 0.8–1.5 mL of sulfur hexafluoride (SF₆) gas. Patients were instructed to maintain a prone position for 1–2 days postoperatively.

### Data collection

The following variables were collected: age, sex, surgical procedure (pars plana vitrectomy alone or combined with cataract surgery), ILM manipulation (inverted ILM flap or peeling), axial length, minimum and basal hole diameters, Gass classification, horizontal OCT-based outer retinal classification, time from OCT examination to surgery, total OR score, location-specific scores (temporal, nasal, superior, inferior) and preoperative and postoperative BCVA at 3 months (logMAR).

### Statistical analysis

Descriptive statistics were calculated for all variables. Spearman’s rank correlation coefficient was used to evaluate the relationships between the total OR score and minimum hole diameter, the total OR score and postoperative BCVA at 3 months, and the total OR score and the time from OCT imaging to surgery.

Multivariable linear regression analysis was performed to identify factors associated with postoperative BCVA at 3 months. Preoperative BCVA and minimum hole diameter were included because they are established predictors of postoperative visual outcomes in eyes with full-thickness macular hole [9, 13]. The interval from OCT imaging to surgery was included to account for potential temporal changes in outer retinal morphology before surgery, and the total OR score was included as the primary variable of interest. To avoid overfitting, the multivariable model was limited to these clinically relevant variables. Variance inflation factors (VIFs) were calculated to assess multicollinearity among the independent variables. To evaluate the incremental prognostic value of the total OR score, multivariable regression models with and without the total OR score were compared using the adjusted coefficient of determination (adjusted R²).

Differences in postoperative BCVA among groups defined by Gass classification and horizontal OCT-based outer retinal classification were assessed using the Kruskal–Wallis test.

Analyses involving postoperative BCVA were performed only in eyes with successful primary macular hole closure. Statistical significance was defined as *p* < 0.05. All statistical analyses were performed using EZR (Saitama Medical Center, Jichi Medical University, Saitama, Japan).

## Results

A total of 48 eyes from 48 patients were included in this study. Four eyes in which primary macular hole closure was not achieved after the initial surgery were excluded from the final analysis of postoperative BCVA at 3 months. Clinical characteristics of these four eyes are summarized in Supplementary Table (1) All four eyes underwent the inverted ILM flap technique. The mean age was 68.8 ± 7.8 years, and 41.7% of patients were male. The mean minimum hole diameter was 386.4 ± 183.6 μm, and the mean basal hole diameter was 933.9 ± 335.6 μm. The mean total OR score was 7.90 ± 1.68. Eight eyes underwent PPV alone, whereas 40 eyes underwent combined phacoemulsification and PPV. Among the eyes treated with PPV alone, seven were pseudophakic and one was phakic preoperatively. No clinically significant cataract progression was observed during the 3-month follow-up. Forty-five eyes (93.8%) underwent the inverted ILM flap technique, whereas three eyes (6.2%) underwent conventional ILM peeling. Descriptive data according to ILM manipulation are presented in Supplementary Table (2) Detailed demographic and clinical characteristics are summarized in Table 1.

**Table 1:** Demographic and clinical characteristics of the study population

Variable	Value
**No. of eyes/patients**	48/48
**Age (years)**	68.8 ± 7.8
**Sex**,** n (%)**	
Male	20 (41.7)
Female	28 (58.3)
**Laterality**,** n (%)**	
Right	19 (39.6)
Left	29 (60.4)
**Axial length (mm)**	24.33 ± 1.89
**Minimum hole diameter (µm)**	386.4 ± 183.6
**Basal hole diameter (µm)**	933.9 ± 335.6
**Preoperative lens status**,** n (%)**	
Phakic	41 (85.4)
Pseudophakic	7 (14.6)
**Surgical procedure**,** n (%)**	
PPV	8 (16.7)
PPV + phaco + IOL	40 (83.3)
**ILM manipulation**,** n (%)**	
Inverted ILM flap technique	45 (93.8)
ILM peeling	3 (6.2)
**Gass classification**,** n (%)**	
Stage 2	8 (16.7)
Stage 3	21 (43.8)
Stage 4	19 (39.6)
**Outer retinal classification (horizontal OCT)**,** n (%)**	
Stage A	5 (10.4)
Stage B	37 (77.1)
Stage C	4 (8.3)
Stage D	2 (4.2)
**Time from OCT imaging to surgery (days)**	23.0 ± 27.5
**Total OR score**	7.90 ± 1.68
Inferior	2.10 ± 0.63
Nasal	1.95 ± 0.55
Superior	2.05 ± 0.64
Temporal	2.08 ± 0.76
**Preoperative BCVA (logMAR)**	0.80 ± 0.37
**Postoperative BCVA at 3 months (logMAR)**	0.36 ± 0.35

PPV, pars plana vitrectomy; Phaco, phacoemulsification; IOL, intraocular lens; ILM, internal limiting membrane; OCT, optical coherence tomography; OR, outer retinal; BCVA, best-corrected visual acuity

Baseline characteristics are based on all 48 eyes. Postoperative BCVA at 3 months is based on the 44 eyes with successful primary macular hole closure after the initial surgery

The weighted Cohen’s kappa coefficient for the total OR score between two graders was 0.90, indicating high interobserver agreement.

**Fig. 1:**
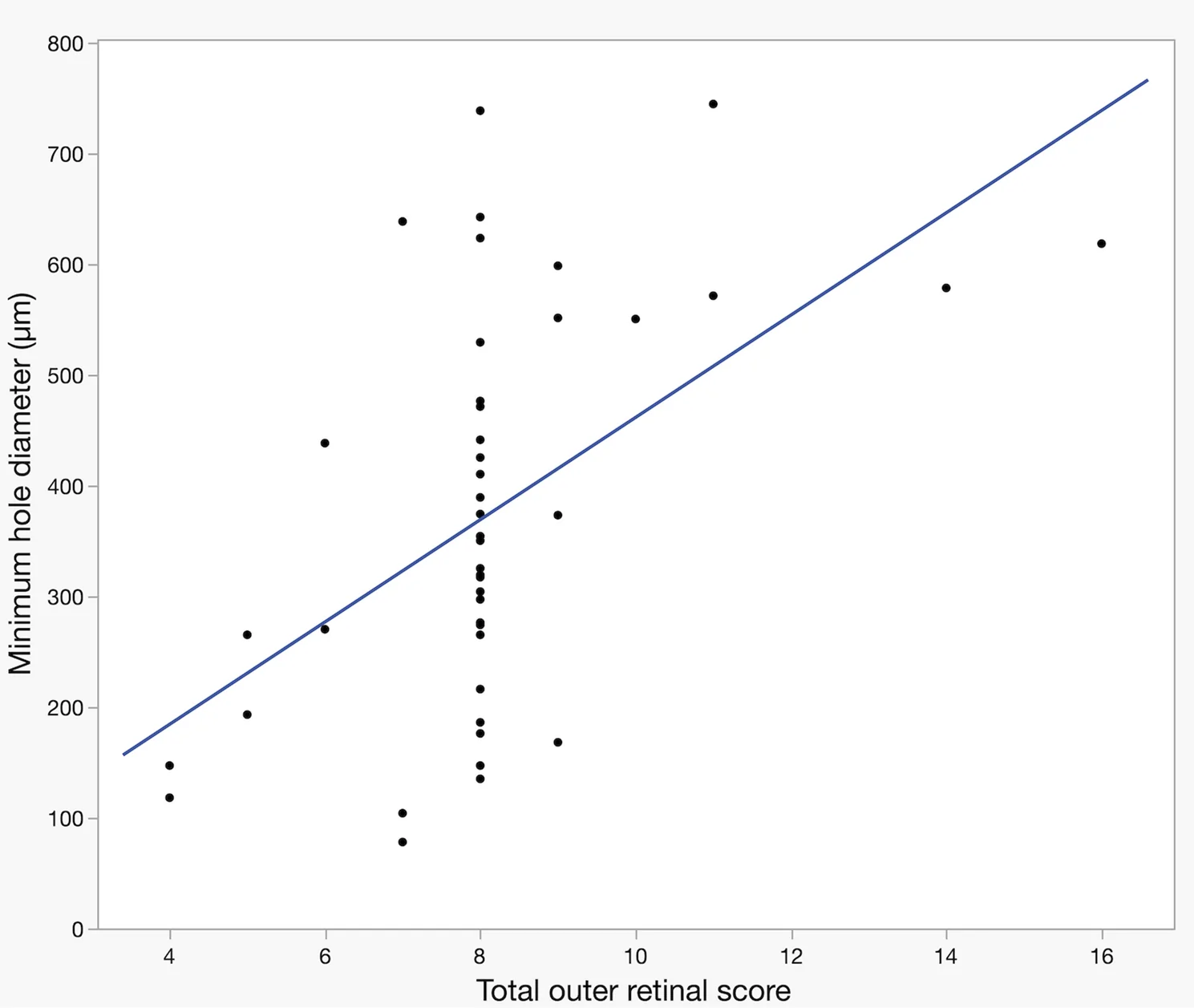
Relationship between the total outer retinal score and minimum hole diameter. Scatter plot showing the association between the total outer retinal (OR) score and minimum hole diameter (μm). Each dot represents an individual eye. The solid line indicates the linear regression line. A significant positive correlation was observed between the total OR score and minimum hole diameter (Spearman’s ρ = 0.522, p < 0.001)

**Fig. 2:**
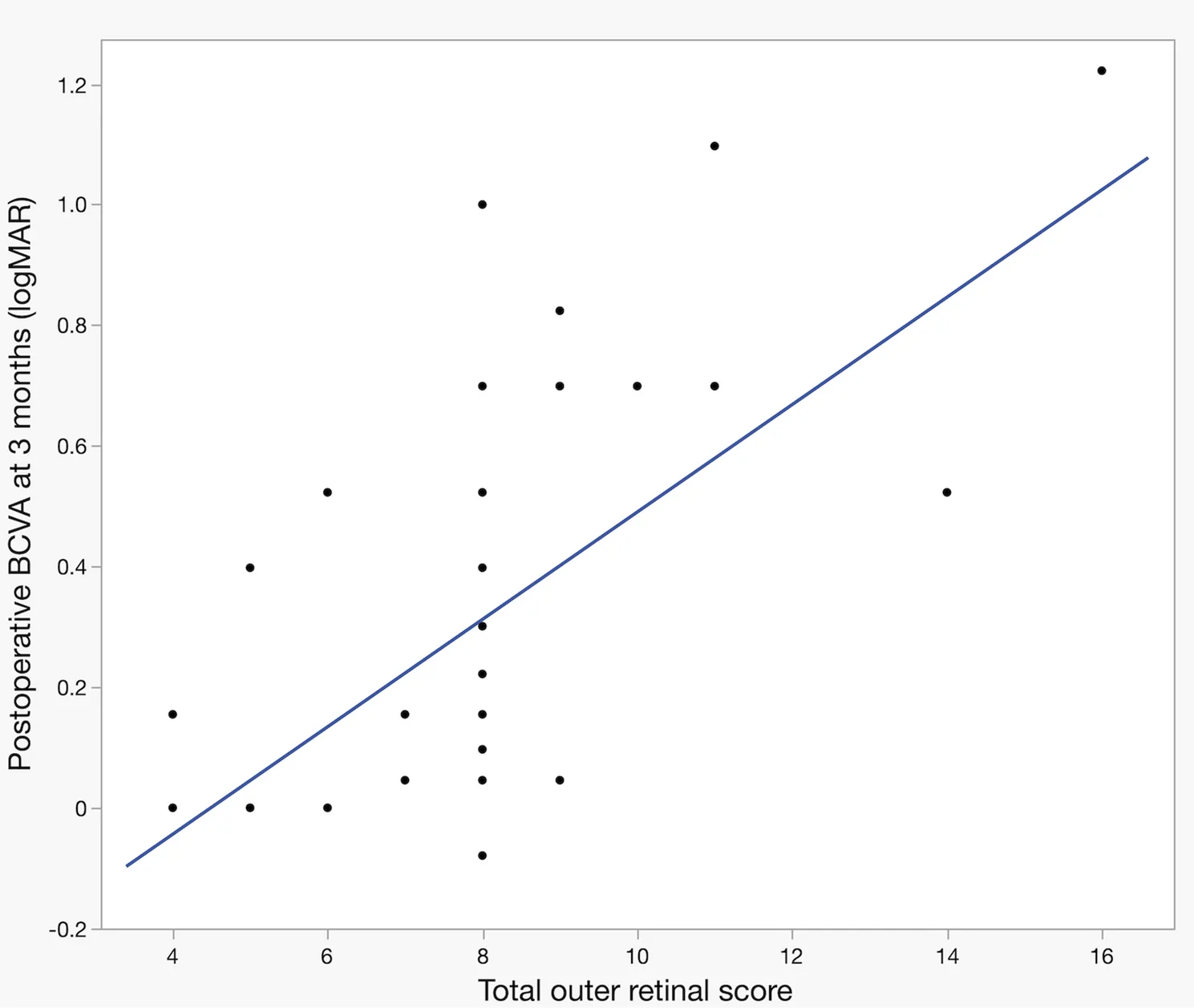
Relationship between the total outer retinal score and postoperative BCVA at 3 months. Scatter plot showing the association between the total outer retinal (OR) score and postoperative best-corrected visual acuity (BCVA) at 3 months (logMAR). Each dot represents an individual eye. The solid line indicates the linear regression line. A significant positive correlation was observed between the total OR score and postoperative BCVA at 3 months (Spearman’s ρ = 0.458, p = 0.0018)

The total OR score showed a significant positive correlation with minimum hole diameter (Spearman’s ρ = 0.522, *p* < 0.001; Fig. 1). A significant correlation was also observed between the total OR score and postoperative BCVA at 3 months (Spearman’s ρ = 0.458, *p* = 0.0018; Fig. 2).

**Fig. 3:**
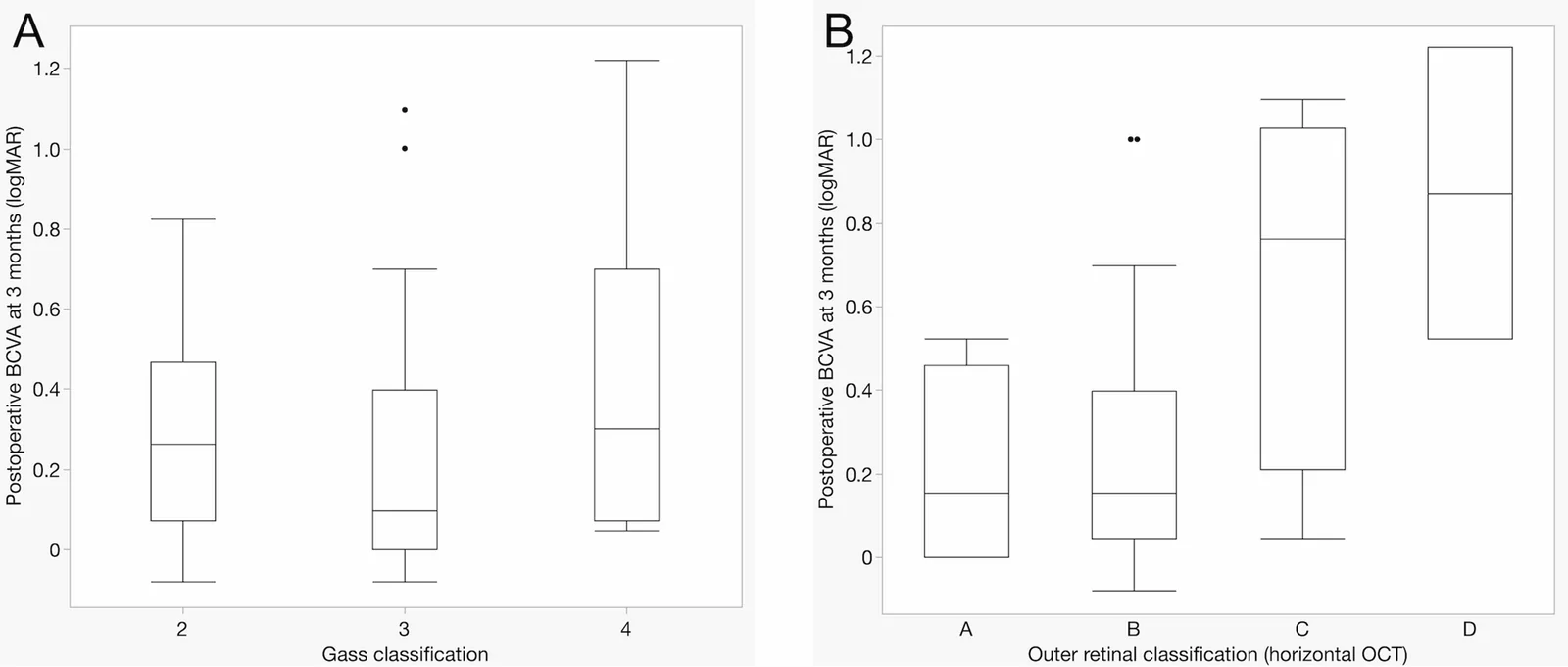
Postoperative BCVA at 3 months according to Gass classification and outer retinal classification (horizontal OCT) (**A**) Box-and-whisker plots showing postoperative best-corrected visual acuity (BCVA) at 3 months (logMAR) according to Gass classification (stages 2, 3, and 4). No significant difference was observed among the groups (Kruskal–Wallis test, p = 0.31). (**B**) Box-and-whisker plots showing postoperative BCVA at 3 months according to outer retinal classification on horizontal optical coherence tomography (OCT) (grades A–D). No significant difference was observed among the groups (Kruskal–Wallis test, p = 0.075). In each box plot, the central line represents the median, the box indicates the interquartile range, and the whiskers represent 1.5 × the interquartile range. Outliers are shown as individual points

Neither the Gass classification nor the horizontal OCT-based outer retinal classification showed a significant association with postoperative BCVA at 3 months (Kruskal–Wallis test, p = 0.31 and p = 0.075, respectively; Fig. 3).

In contrast, no significant correlation was found between the time from OCT imaging to surgery and the total OR score (Spearman’s ρ = 0.082, *p* = 0.60). Outer retinal findings were not identical across all four locations in 33% of cases, suggesting spatial heterogeneity.

In the multivariable regression analysis, minimum hole diameter (β = 0.061 per 100 μm increase, 95% CI: 0.014–0.108, *p* = 0.013) and preoperative BCVA (β = 0.479, 95% CI: 0.259–0.699, *p* < 0.001) were independently associated with postoperative BCVA at 3 months. In contrast, the total OR score (β = 0.019, 95% CI: −0.025 to 0.063, *p* = 0.387) and the time from OCT imaging to surgery (β = 0.0017, 95% CI: −0.0010 to 0.0044, *p* = 0.198) were not independently associated with postoperative BCVA at 3 months (Table 2). Variance inflation factors ranged from 1.10 to 1.73, indicating no evidence of problematic multicollinearity among the independent variables. Comparison of multivariable models with and without the total OR score showed that inclusion of the total OR score did not improve model performance (adjusted R²: 0.556 without the OR score vs. 0.554 with the OR score).

**Fig. 4:**
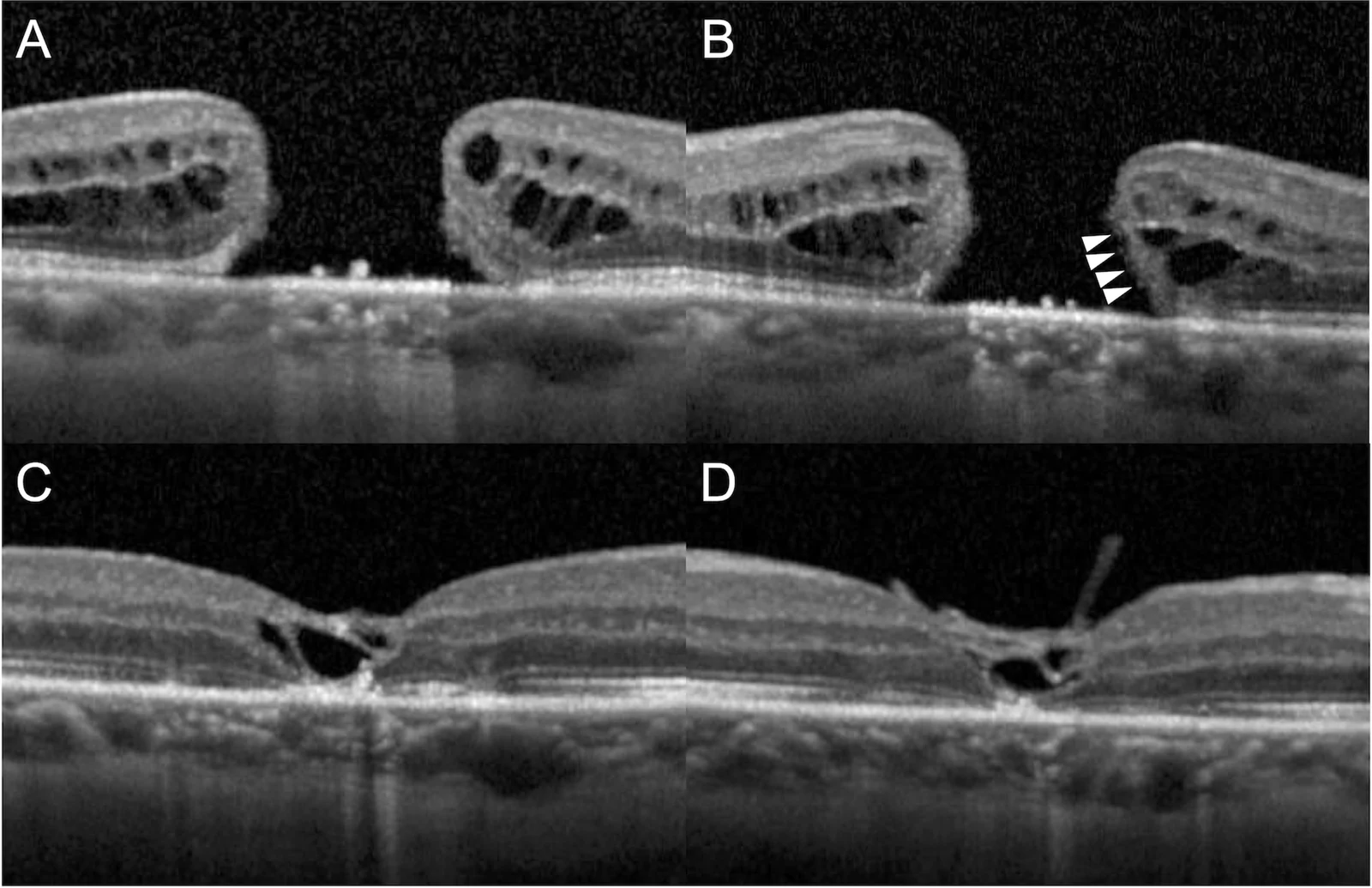
Preoperative and postoperative OCT images of a representative case. (**A**, **B**) Preoperative optical coherence tomography (OCT) images. (**C**, **D**) OCT images obtained 3 months postoperatively. (**A**) Horizontal scan of the right eye. The left side corresponds to the temporal retina, and the right side corresponds to the nasal retina. The outer retinal layers at both the temporal and nasal edges of the macular hole show swelling, corresponding to outer retinal classification stage B. Intraretinal cystoid spaces are observed in both the inner nuclear layer (INL) and outer nuclear layer (ONL). (**B**) Vertical scan. The left side corresponds to the inferior retina, and the right side corresponds to the superior retina. The outer retina at the inferior edge shows swelling consistent with stage B, whereas the superior edge demonstrates a moth-eaten appearance (white triangle) of the outer retinal layers, corresponding to stage C. (**C**, **D**) Postoperative OCT images at 3 months. (**C**) Horizontal scan. (**D**) Vertical scan.The macular hole is closed. The ellipsoid zone remains disrupted at the fovea and is replaced by hyperreflective tissue. The inverted internal limiting membrane flap is observed bridging over the fovea within the inner retinal layers

**Table 2:** Multivariable regression analysis of factors associated with postoperative BCVA at 3 months

Parameters	β (95% CI)	*p* Value
Time from OCT imaging to surgery (days)	0.0017 (-0.0010, 0.0044)	0.198
Minimum hole diameter (per 100 μm)	0.061 (0.014, 0.108)	0.013
Total OR score	0.019 (-0.025, 0.063)	0.387
Preoperative BCVA (logMAR)	0.479 (0.259, 0.699)	< 0.001

OCT, optical coherence tomography; OR, outer retinal; BCVA, Best-corrected visual acuity

## Representative case

A 59-year-old woman presented with a full-thickness macular hole in her right eye. The preoperative BCVA was 20/250.

Preoperative OCT revealed a hyperreflective homogeneous thickened band in the outer retinal layers at the edges of the macular hole at the temporal, nasal, and inferior sides, corresponding to outer retinal classification stage B, whereas the superior edge demonstrated a moth-eaten appearance of the outer retinal layers, corresponding to stage C. The total OR score was 9 in this case (Fig. 4A, B).

She underwent combined phacoemulsification and pars plana vitrectomy with the ILM flap technique.

At 3 months postoperatively, the macular hole was successfully closed. Her BCVA improved to 20/100 at 3 months after surgery. Postoperative OCT demonstrated bridging tissue at the fovea corresponding to the inverted ILM flap within the inner retinal layers. The EZ and ELM remained disrupted at the fovea and were replaced by hyperreflective material (Fig. 4C, D).

## Discussion

The total OR score, derived from four-location assessment of the macular hole edge, was significantly associated with postoperative BCVA at 3 months in univariate analysis. However, this association was not maintained in the multivariable analysis, which included minimum hole diameter. Furthermore, comparison of multivariable models with and without the total OR score demonstrated virtually identical model performance. These findings suggest that the total OR score does not provide incremental prognostic value beyond established predictors, particularly minimum hole diameter. Unlike the conventional single horizontal OCT classification, which was not significantly associated with postoperative BCVA, the total OR score showed a significant association in univariable analysis. Although this association did not remain independent after multivariable adjustment, evaluation using both horizontal and vertical scans enabled standardized assessment of four macular hole edge locations and may provide a more comprehensive description of outer retinal morphology than evaluation based on a single horizontal scan. Accordingly, the total OR score may be useful as a standardized method for describing the severity and spatial distribution of outer retinal morphological changes by evaluating four locations around the macular hole. The total OR score showed a significant correlation with minimum hole diameter, suggesting that both parameters capture overlapping aspects of morphological severity.

Minimum hole diameter has been established as a key predictor of postoperative visual outcomes [9] and the observed correlation between the total OR score and hole diameter may explain the loss of independent significance in multivariable analysis. In contrast, neither the Gass classification nor single cross-sectional classification based on horizontal OCT scans showed a significant association with postoperative BCVA in this study.

The Gass classification has been widely used to describe the progression of macular hole formation [1]. However, with advances in surgical techniques, including ILM peeling and the inverted ILM flap technique, high closure rates can now be achieved even in large macular holes [5–7]. Accordingly, clinical focus has shifted from anatomical closure to functional outcomes, and the Gass classification may be insufficient as a predictor of postoperative BCVA.

Recent studies have demonstrated that outer retinal structures, such as the ELM and EZ, are closely associated with postoperative visual function [11–14]. Pecaku et al. adapted a classification of outer retinal morphology originally described in rhegmatogenous retinal detachment and applied it to macular hole edges [15–17]. Although this classification was associated with postoperative BCVA, it was based on a single cross-sectional image and may not fully capture spatial heterogeneity of outer retinal disruption.

In the present study, we extended this concept by evaluating outer retinal morphology in four locations (temporal, nasal, superior, and inferior) using both horizontal and vertical OCT scans, and integrated these findings into the total OR score. This four-location approach may allow a more comprehensive assessment of outer retinal damage that cannot be captured by a single cross-sectional image. The significant association between total OR score and postoperative BCVA suggests that this parameter may reflect morphological severity.

Outer retinal morphology has been reported to change over time; however, in this study, no significant correlation was observed between the interval from OCT imaging to surgery and the total OR score, suggesting that the impact of changes over time was limited.

This study has several limitations. It was a retrospective, single-center study with a relatively small sample size. Although ILM manipulation was not fully standardized, only three eyes underwent conventional ILM peeling. Because of this highly unbalanced distribution, formal statistical comparison between surgical techniques and inclusion of ILM manipulation in the multivariable model were not considered appropriate. Therefore, the potential influence of surgical technique on postoperative visual outcomes cannot be excluded and should be evaluated in future studies including a larger number of eyes treated with conventional ILM peeling. Eyes without primary closure were excluded from the postoperative BCVA analysis, which may have introduced selection bias. However, because all four eyes without primary closure underwent the inverted ILM flap technique, differences in ILM manipulation are unlikely to explain this potential bias. Most eyes underwent combined phacoemulsification and vitrectomy. Because only one eye undergoing PPV alone was phakic and no clinically significant cataract progression was observed during follow-up, the influence of cataract progression on postoperative BCVA was likely minimal. Nevertheless, the potential confounding effect of combined phacoemulsification cannot be completely excluded. OCT evaluation was based on selected horizontal and vertical B-scans rather than full OCT volume scans, which may not fully represent the entire macular structure. The follow-up period was limited to 3 months. BCVA and restoration of the outer retinal layers, including the ELM and EZ, may continue to improve for up to 12 months after macular hole surgery [10, 11]. Therefore, the relatively short follow-up period in the present study may have underestimated the prognostic value of the total OR score, and its association with longer-term visual outcomes should be investigated in future studies. Further large-scale prospective studies are warranted.

In conclusion, the total OR score was associated with postoperative BCVA in univariate analysis, but did not demonstrate independent prognostic value after multivariable adjustment for established prognostic factors. The score may reflect outer retinal damage associated with hole size.

## Supplementary Information

Below is the link to the electronic supplementary material.

Supplementary Material 1

Supplementary Material 2

## Data Availability

The datasets generated and/or analyzed during the current study are available from the corresponding author on reasonable request.
