# Supplementary material for: Association between a novel four-location outer retinal morphology score and visual outcomes in full-thickness macular hole
Source: Int J Retina Vitreous. 2026 Jul 31;12:144. doi: 10.1186/s40942-026-00912-4 (PMC13715197; doi:10.1186/s40942-026-00912-4)
Supplement: Supplementary file 1 [file 40942_2026_912_MOESM1_ESM.docx]

Supplementary Table 1

Clinical characteristics of eyes without primary macular hole closure

| **Eye** | **Minimum hole diameter (μm)** | **Total OR score** | **ILM manipulation** |
| --- | --- | --- | --- |
| 1 | 550 | 8 | Inverted ILM flap technique |
| 2 | 272 | 8 | Inverted ILM flap technique |
| 3 | 647 | 8 | Inverted ILM flap technique |
| 4 | 616 | 8 | Inverted ILM flap technique |

OR, outer retinal; ILM, internal limiting membrane
