# Supplementary material for: Association between a novel four-location outer retinal morphology score and visual outcomes in full-thickness macular hole
Source: Int J Retina Vitreous. 2026 Jul 31;12:144. doi: 10.1186/s40942-026-00912-4 (PMC13715197; doi:10.1186/s40942-026-00912-4)
Supplement: Supplementary file 2 [file 40942_2026_912_MOESM2_ESM.docx]

Supplementary Table 2

Clinical characteristics and postoperative outcomes according to ILM manipulation

| **ILM manipulation** | **n** | **Minimum hole diameter (μm)** | **Total OR score** | **Primary closure, n (%)** | **Postoperative BCVA at 3 months (logMAR)** |
| --- | --- | --- | --- | --- | --- |
| Inverted ILM flap | 45 | 402.0 ± 179.0 | 8.2 ± 2.0 | 41 (91.1) | 0.37 ± 0.35 |
| ILM peeling | 3 | 152.7 ± 21.1 | 6.7 ± 2.3 | 3 (100.0%) | 0.21 ± 0.20 |

ILM, internal limiting membrane; OR, outer retinal; BCVA, best-corrected visual acuity.
